# Insulin initiation in patients with type 2 diabetes is often delayed, but access to a diabetes nurse may help—insights from Norwegian general practice

**DOI:** 10.1080/02813432.2023.2296118

**Published:** 2024-02-07

**Authors:** Ibrahimu Mdala, Kjersti Nøkleby, Tore Julsrud Berg, John Cooper, Sverre Sandberg, Karianne Fjeld Løvaas, Tor Claudi, Anne Karen Jenum, Esben Selmer Buhl

**Affiliations:** a Department of General Practice, Institute of Health and Society, University of Oslo (UiO), Norway; b Institute of Clinical Medicine, University of Oslo (UiO), Norway; c Department of Endocrinology, Oslo University Hospital (OUS), Norway; d Norwegian Quality Improvement of Laboratory Examinations, Haraldsplass Deaconess Hospital, Bergen (HDS), Norway; e Division of Medicine, Stavanger University Hospital (SUS), Norway; f Department of Global Public Health and Primary Care, University of Bergen (UiB), Norway; g Clinic For Medicine, Nordland Hospital, Bodø, Norway

**Keywords:** Type 2 diabetes mellitus, general practice, primary care, therapeutic inertia, basal insulin, insulin initiation, diabetes nurse

## Abstract

**Objective**: We opted to study how support staff operational capacity and diabetes competences may impact the timeliness of basal insulin-initiation in general practice patients with type 2 diabetes (T2D).

**Design/Setting/Outcomes:** This was an observational and retrospective study on Norwegian primary care patients with T2D included from the ROSA4-dataset. Exposures were (1) support staff size, (2) staff size relative to number of GPs, (3) clinic access to a diabetes nurse and (4) share of staff with diabetes course (1 and 2 both relate to staff operational capacity, whereas 3 and 4 are both indicatory of staff diabetes competences). Outcomes were ‘timely basal insulin-initiation’ (primary) and ‘attainment of HbA_1c_<7%’ after insulin start-up (secondary). Associations were analyzed using multiple linear regression, and directed acyclic graphs guided statistical adjustments.

**Subjects:** Insulin naïve patients with ‘timely’ (*N* = 294), ‘postponed’ (*N* = 219) or ‘no need of’ (*N* = 3,781) basal insulin-initiation, respectively.

**Results:** HbA_1c_ [median (IQR)] increased to 8.8% (IQR, 8.0, 10.2) prior to basal insulin-initiation, which reduced HbA_1c_ to 7.3 (6.8–8.1) % by which only 35% of the subjects reached HbA_1c_ <7%. Adjusted risk of ‘timely basal insulin-initiation’ was more than twofold higher if access to a diabetes nurse (OR = 2.40, [95%CI, 1.68, 3.43]), but related only vaguely to staff size (OR = 1.01, [95%CI, 1.00, 1.03]). No other staff factors related significantly to neither the primary nor the secondary outcome.

**Conclusion:** In Norwegian general practice, insulin initiation in people with T2D may be affected by therapeutic inertia but access to a diabetes nurse may help facilitating more timely insulin start-up.

## KEY POINTS

- In patients with type 2 diabetes (T2D) cared for by their general practice physician (GP), insulin therapy was susceptible to therapeutic inertia.
- In Norwegian general practice, chance of timely basal insulin-initiation was found more than two-fold higher if the GP had access to a diabetes nurse.
- In contrast, the timeliness of basal insulin-initiation in general practice patients with T2D seemed unaffected by share of support staff with diabetes course and by factors indicatory of support staff overall operational capacity.
- In Norwegian general practice, a diabetes nurse seems to offer unique clinical benefits to the care of insulin treated patients with T2D.

## Introduction

Therapeutic inertia, defined as failure of health-care provider to initiate or intensify therapy when therapeutic targets are not attained, is well-known in relation to treatment of most chronic diseases [1,2]. Although therapeutic inertia seems to affect all elements of the treatment cascade for type 2 diabetes (T2D), insulin initiation and intensification are still the key treatment steps most often affected [3–7]. The resulting consequences are hyperglycemia and increased risk of long-term complications [8].

Several barriers to timely and adequate insulin therapy may exist [9]. Barriers may be attributed to the organization of the provided care, to doctor competences and attitudes, and to patient beliefs and perceptions [5]. Firstly, resource constraints, such as limited staff support or little time per consultation, are key organizational factors that may cause postponement of more complex and time-consuming treatment measures such as insulin initiation. Secondly, the doctor’s attitude regarding when to start insulin [10], or his/her clinical experience and competence with tackling key challenges related to insulin treatment, such as technical operation of insulin pens, hypoglycemia, weight gain and/or nonadherence, are also factors to consider [5,10,11]. Thirdly, people with T2D may themselves be reluctant to start insulin therapy due to beliefs, perceptions and/or myths. Skepticism toward the safety (e.g. fear of needles, hypoglycemia and/or weight gain) or efficacy of insulin, or the notion that insulin may result in social stigma or may be a punishment for failing self-care, are examples of potential beliefs involved when individuals with T2D oppose insulin [5,11–13].

In Norway, general practice provides care to the vast majority of people with T2D, and initiating insulin is often recognized as the most challenging step in the diabetes treatment cascade [7]. According to national guidelines [14], most Norwegian general practitioner physicians (GPs) will initiate insulin therapy by adding basal insulin on top a regimen of non-insulin anti-diabetic drugs (NIADs) which may or may not be modified prior to insulin start-up. Norwegian reimbursement restrictions stipulate NPH insulin (e.g. Neutral Protamine Hagedorn (NPH)) as the mandatory first choice when initiating basal insulin in people with T2D. If glycemic targets still are not reached, the GP can consider a pre-mixed insulin formulation or administering a meal-time insulin. If hypoglycemia is detected, longer duration basal insulin-analogues, offering lower day-to-day glycemic variation and lower risk of hypoglycemia [15,16], may be considered. In Norwegian general practice, however, still little is known about the quality of basal insulin-initiation, for example, to which extent the GP manages to initiate timely basal insulin therapy and to attain the glycemic target after starting insulin treatment.

Healthcare teams with a diabetes nurse have been suggested to ensure that Norwegian primary care is ready to tackle the increasing prevalence of T2D [17]. In Norwegian general practice, however, only relatively few clinics have a diabetes nurse due to the costs, and little is known about how access to a diabetes nurse or support staff with hands-on competencies for following up individuals with diabetes may affect the quality of basal insulin-initiation.

In preliminary analyses (Supplementary Table 1), we found GP demographic factors to be rather unrelated to the quality of basal insulin initiation in people with T2D who are treated and cared for in Norwegian general practice. So instead, in subjects with T2D eligible for insulin start-up, we aimed to assess possible associations between indicators of support staff operational capacity and support staff diabetes competences, including GP access to a diabetes nurse, (exposures) and the risk of *timely* vs. *postponed* basal insulin initiation (primary outcome) and the chances of attaining HbA_1c_ <7% (53 mmol/mol) subsequent to insulin initiation (secondary outcome).

## Methods

### Data source

We employed population-based cross-sectional data from year 2014 from the ROSA 4-study on the care of people with diabetes in general practice. ROSA 4 involves five counties in Norway, a total 77 practices, 282 GPs, and 11,428 subjects aged ≥ 18 years with T2D [18,19]. ROSA 4, supplemented with data on ethnicity, provides us with data on patient clinical features, GP demographics, practice clinic organization characteristics, including indicators of clinic support staff involvement in diabetes follow-up. For the latter, we use the phrase ‘ROSA4-hands-on diabetes follow-up tasks’ (more details given in Supplementary Table 3B), and all of these are dichotomous categorical variables stating whether or not (yes or no) support staff performs a given follow-up hands-on task with either possible immediate/direct impact on glucose control and study outcomes, for example, (1) patient 1-on-1 counselling in diet, (2) self-measurement of blood glucose, (3) self-injection of insulin and/or GLP-1-RA and (4) systematic usage of the NOKLUS^1^ form, or without possible direct effect on glycemic control or study outcomes, for example, (1) performance of 1-on-1 patient foot care and (2) other less well-defined tasks in relation to organizing and executing the annual diabetes control (share of GP clinics with support staff performing various follow-up tasks are shown in the lower panel of Supplementary Table 2). The term ‘diabetes nurse’ is defined as a nurse with some form of postgraduate diabetes training and/or education (Supplementary Figure 1). Also, the present dataset offers the numerical variable ‘staff-diabetes course’ which provides the share of support staff (e.g. a continuous variable between 0 to 100%) who within the last 3 years have participated in a diabetes course (Supplementary Table 3A). While all of the former variables are cross-sectional, in addition ROSA 4 also offers longitudinal data on HbA_1c_ and drug prescriptions, primarily for the years 2012–2014 (e.g. 3 years), although HbA_1c_ sometimes was recorded with additional measurements in the years 2015–2016 (e.g. up to additional 2 years).

### Identification of the three main study groups

**Figure 1.**
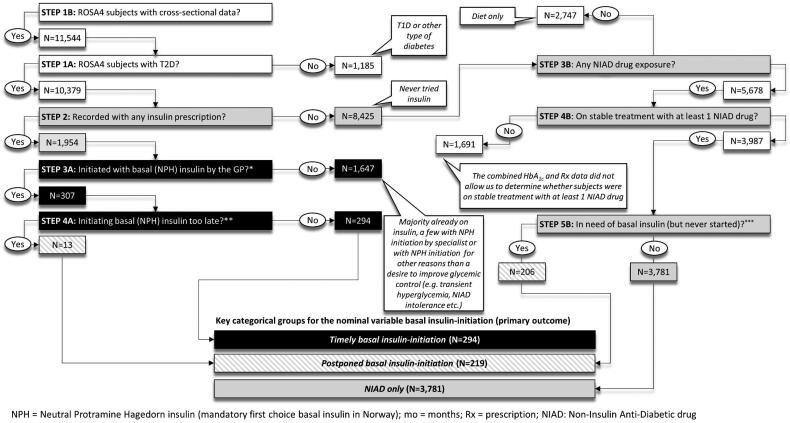
Selection algorithm for identifying ROSA4 participants with type 2 diabetes (T2D) for the three categories of the primary outcome nominal variable.

Among 11,544 ROSA 4-subjects, we identified individuals with T2D and potentially eligible to start on basal insulin: (1) Subjects with *timely* basal (NPH) insulin-initiation (*n* = 294), (2) subjects with *postponed* basal (NPH) insulin-initiation (*n* = 219), and subjects with (3) *NIAD only* treatment (*n* = 3,781). The latter served as a reference group in the regression analyses in relation to the primary outcome (see below). Patients with T2D who were not on stable treatment with noninsulin anti-diabetic drugs (NIADs), including those on only lifestyle and/or diet intervention only, or who were already on insulin, were not considered eligible for insulin initiation and hence excluded. The postponed insulin-initiation group consisted of both people with incident but too late insulin exposure (*n* = 13) (e.g. 4.2% of all basal insulin initiators (*n* = 307)), and of insulin-naïve people with an urgent need of insulin initiation which had lasted for at least 180 days without insulin initiation ever being performed (e.g. 4.9% of all NIAD only treated, *n* = 206). Figure 1 provides the algorithm and the criteria by which we identified and included subjects to the three study categories. Attainment of HbA_1c_<7% (53 mmol/mol) (e.g. secondary outcome) was studied in basal insulin-initiators with complete follow-up data for up to 33 months (median follow-up time was 14 (IQR, 9, 19) months) after first insulin (NPH) prescription (*n* = 248).

### Clinical context of the ROSA4 dataset and the current study

**Figure 2.**
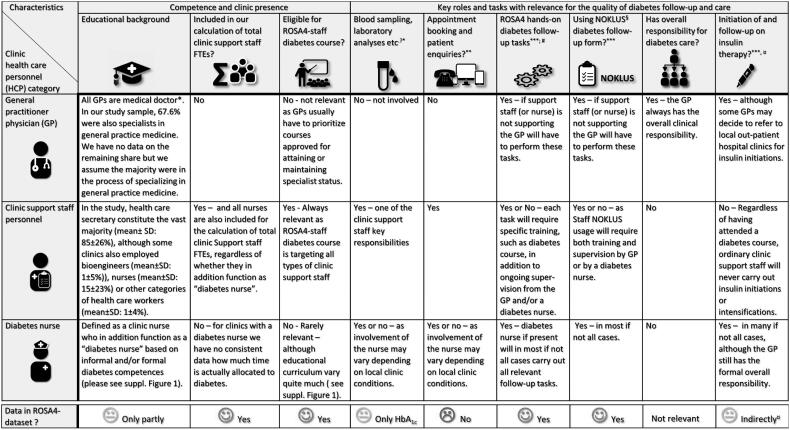
Descriptive overview of key health care professionals and their roles and tasks in relation to diabetes follow-up in our Norwegian general practice study.

In Norway, most GPs work in smaller clinics with on average about four GPs per clinic (Supplementary Table 2) together with support staff, whereof the vast majority are educated health care secretaries (e.g. 85% in our sample, Figure 2). Most general practices in Norway have about 0.8 support staff personnel per GP (Supplementary Table 2), where a lower ratio might indicate a higher workload for the support staff. Moreover, the clinic size itself may also affect the support staff operational capacity as larger clinics with more support staff may benefit from ‘*economies of scale’* as compared to smaller clinics [20]. The degree to which the support staff is (hands-on) involved in diabetes care may also vary between clinics (e.g. the ROS4-hands-on diabetes follow-up tasks which the support staff may or may not perform). Figure 2 details different categories of general practice personnel and how they may be involved hands-on in diabetes care, and Supplementary Table 2 shows the share of GP clinics with support staff performing diabetes follow-up tasks. In some GP clinics, support staff members are further provided with a diabetes course to augment their diabetes care competence (Figure 2). A few clinics have access to a diabetes nurse, either employed as support staff by the clinic or made available to the clinic by the local municipal primary health care organization (21.6% of clinics in the current sample had access to a diabetes nurse (Supplementary Table 2)). A diabetes nurse typically has both relevant clinical experience and some form of post-graduate education in the field of diabetes care. Supplementary Figure 1 provides an overview of the typical curriculum as well as key deliverables for a primary care diabetes nurse in Norway.

### Data analysis

For the analyses, patients were nested within their GPs, who in turn were nested within their practices at levels two and three respectively. Then, data on basal insulin initiation (primary outcome), for example, a nominal variable with 3 categories (1. *Postponed* basal insulin-initiation 2. *Timely* basal insulin initiation, and 3. *NIAD only treated*), were explored using a multinomial generalized structural equation model (GSEM) accounting for shared variance of patients and GPs nested within their GP and practices, respectively. Secondly, a multilevel binary logistic regression model was employed to assess the binary outcome in the total sample of basal insulin-initiators with complete follow-up (*n* = 248), for example, attainment of HbA_1c_<7% (53 mmol/mol) (yes/no) (secondary outcome). Firstly, we assessed two exposures indicative of support staff diabetes competences, that is, (1*) ‘clinic diabetes nurse’* (categorical variable, yes/no) and (2) *‘share of support staff with diabetes course’* (continuous variable) (Supplementary Table 3A). Secondly, we analyzed the effect of two additional exposures more related to the overall operational capacity of the support staff [20], i.e., (1) *‘staff size’*, a continuous variable expressed in *full-time equivalents (FTEs^2^),* and (2) *‘support staff work-load’,* a continuous variable, computed as the *support staff-to-GP FTE–ratio,* that provides the total number of support staff employees relative to the total number of GPs (Supplementary Table 3A). In contrast, support staff hands-on diabetes follow-up tasks (i.e. support staff responsible for using the structured national follow-up tool ‘Noklus diabetes form’, for providing diet counseling, and/or for training patients in blood glucose (BG) measurements and/or injections (Supplementary Table 3B)) where considered as only mediators. Other practice characteristics as well as patient factors were all, based on drawn directed acyclic graphs, identified as ‘non-confounders for the outcomes’, and hence were not considered in the adjusted analyses. For all analyses, we considered only complete/observed data, assuming missing by random. Multiple regression analyses of defined outcomes and exposures were guided by directed acyclic graphs (DAGs) drawn by use of the online freeware DAGitty 3.0 at http://dagitty.net. Computed risks of outcomes are given as relative risk ratios (RRRs) or odds ratios (ORs), respectively, with 95% confidence intervals (CIs).

Categorical variables were assessed by Chi-square tests if expected cell counts were > 5, if not by Fisher’s exact. Continuous variables are given as means ± SD or medians with interquartile range (IQR). Differences in normally distributed continuous variables were explored using analysis of variance (if three groups) or independent samples T-test (if two groups). Non-normally distributed continuous variables were assessed by Kruskal–Wallis test (if three groups) or Mann–Whitney U-test (if two groups). StataSE 17 was used for statistical analyses. Significance level was set at α = 0.05.

## Results

Descriptive characteristics of the three groups potentially eligible for basal insulin initiation are given in Table 1. Individuals with basal-insulin initiation, as compared to *NIAD only treated*, were a bit younger, had longer diabetes duration and higher levels of HbA_1c_. Also, subjects with *postponed* as compared to *timely* basal insulin-initiation, despite being a little younger, had higher HbA_1c_, used more NIADs, were less frequently females and more often had Asian ethnical background. At least one macro-vascular complication was more frequent in patients with *timely* basal insulin initiation, and at least one microvascular was more abundantly found in both patients *timely* and *postponed* basal insulin initiation, although highest frequency was seen in subjects with *timely* basal insulin initiation.

**Table 1.** Descriptive analysis of the characteristics of subjects with type 2 diabetes included into the three nominal study groups employed in primary outcome analysis.

Study groups Key descriptive factors	*Timely* basal insulin-initiation			*Postponed basal* insulin-initiation			***NIAD only*** * (reference group)*
	Covariate	RRR (95%CI)	*P*-value	Covariate	RRR (95%CI)	*P*-value	Covariate
*n* (%)	294 (6.8)	–	–	219 (5.1)	–	–	3781 (88.0)
***Demographics***							
Age (Mean ± SD)	63.6 ± 14.1	0.99 (0.98–1.00)	< 0.03	61.4 ± 12.1	0.98 (0.96–.099)	< 0.01	64.9 ± 12.4
Gender: n (%)							
Males	167 (57.8)	Ref.	–	137 (63.5)	Ref.	–	2053 (54.9)
Females	124 (42.2)	0.89 (0.73–1.08)	0.37	80 (36.5)	0.73 (0.54–.098)	0.04	1685 (45.1)
Ethnicity: n (%)							
European	254 (86.4)	Ref	–	173 (79.0)	Ref	–	3187 (84.3)
African	5 (1.7)	1.03 (0.40–2.63)	0.96	2 (0.9)	0.66 (0.16–2.77)	0.57	64 (1.7)
Asian	32 (10.9)	0.77 (0.51–1.15)	0.20	42 (19.1)	1.56 (1.06–2.30)	0.02	492 (13.0)
Other or unknown	3 (1.0)			2 (0.9)	–	–	38 (1.0)
Diabetes duration (years)	11.0 (6.0, 16.2)	1.07 (1.05–1.09)	< 0.01	10.0 (7.0, 15.0)	1.06 (1.04–1.08)	< 0.01	8.0 (5.0, 12.0)
***Disease control efforts and status***							
Sum of NIADS	1.0 (0.0, 1.0)	0.31 (0.25, 0.37)	< 0.01	2.0 (2.0, 3.0)	3.60 (3.04, 4.29)	< 0.01	1.0 (1.0, 2.0)
HbA_1c_ measurements per year*	3.67 (2.33; 4.67)	1.14 (1.11; 1.16)	<0.01	2.67 (2.00; 4.00)	1.01 (1.04; 1.07)	0.02	2.67 (1.67; 3.67)
HbA_1c_ (% [mmol/mol])	8.8 (8.0, 10.2) [73 (64, 88)]	1.64 (1.55, 1.74)	< 0.01	10.0 (9.0,14.0) [86 (75, 130)]	1.91 (1.79, 2.03)	< 0.01	6.9 (6.3, 7.5)
BMI (kg/m^2^)	31.0 (26.6, 35.6)	1.06 (1.03, 1.08)	< 0.01	30.7 (26.1, 33.5)	1.04 (1.00, 1.07)	0.02	29.1 (26.0, 32-8)
Systolic Blood Pressure (mmHg)	136 (125, 146)	1.00 (1.00, 1.01)	0.19	135 (125, 144)	1.00 (0.99, 1.01)	0.66	134 (125, 144)
Diastolic Blood Pressure (mmHg)	80 (70, 85)	1.00 (0.99, 1.02)	0.54	80 (75, 85)	1.02 (1.00, 1.04)	0.02	80 (71, 83)
Total cholesterol (mmol/l)	5.1 (4.3, 6.1)	1.05 (0.98, 1.12)	0.17	5.0 (4.2, 6.0)	1.01 (0.94, 1.10)	0.75	5.0 (4.3, 5.9)
LDL cholesterol (mmol/l)	3.0 (2.4, 3.9)	1.00 (0.92, 1.10)	0.94	3.0 (2.3, 3.8)	1.01 (0.91, 1.12)	0.91	3.1 (2.4, 3.8)
HDL cholesterol (mmol/l)	1.2 (1.0, 1.5)	0.79 (0.62, 0.99)	0.05	1.1 (1.0, 1.4)	0.84 (0.63, 1.10)	0.21	1.3 (1.1, 1.5)
Triglycerides (mmol/l)	2.5 (1.8, 3.9)	1.11 (1.07, 1.15)	< 0.01	2.4 (1.7, 3.4)	1.07 (1.02, 1.13)	< 0.01	2.0 (1.4, 2.8)
Creatinine (μmol/l)	91 (75, 125)	1.01 (1.01, 1.02)	< 0.01	83 (71, 94)	1.00 (1.00,1.01)	0.87	80 (68, 95)
***Complications***							
*Microvascular diseases, (n, %)*							
None	194 (66.0)	Ref	–	176 (80.4)	Ref	–	3354 (88.7)
One or more	100 (34.0)	4.09 (3.11–5.39)	<0.01	43 (19.6)	1.98 (1.36–2.88)	<0.01	427 (11.3)
*Macrovascular diseases, (n, %)*							
None	182 (61.9)	Ref	–	161 (73.5)	Ref	–	2790 (73.8)
One or more	112 (38.1)	1.66 (1.29–2.14)	<0.01	58 (26.5)	0.97 (0.70–1.35)	0.88	991 (26.2)

Unadjusted relative risk ratios (RRR) with 95% CIs obtained from logistic regression. Relative risk ratios for the third nominal outcome, NIAD only, are not shown. Continuous variables are given as Medians, IQR. Differences between categorical variables, given as percentages. *Norwegian guidelines recommended (e.g. year 2012–2014) and still recommend (e.g. year 2023) at least one annual diabetes control for each patient, and eventually up to one to three additional follow-up controls based on an individual clinical assessment of each patient. All GPs and diabetes nurses are obliged to follow this recommendation.

**Figure 3.**
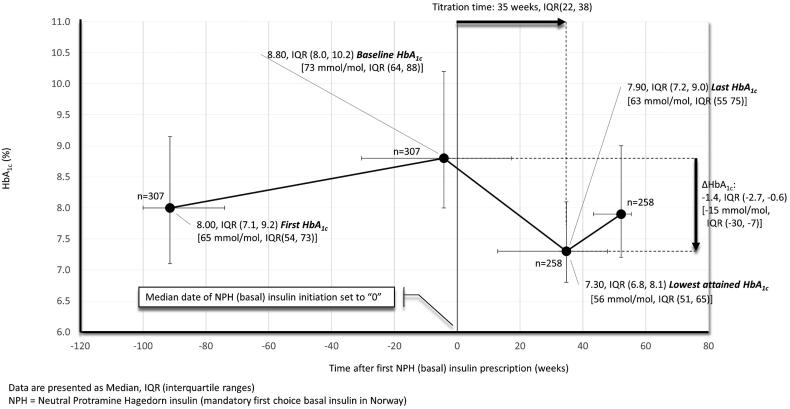
HbA_1c_ development in NPH (basal insulin) initiators.

Figure 3 shows median HbA_1c_ levels in basal insulin initiators before and after insulin start-up. With basal insulin initiation, median HbA_1c_ declined from 8.8% (IQR, 8.0, 10.2) (73 mmol/mol (IQR, 64, 88)) at baseline to 7.3% (IQR, 6.8, 8.1) (56 mmol/mol (IQR, 51, 65)) after a median titration time of 34 weeks (IQR, 22, 38). Further, the rather high baseline HbA_1c_ gave an impression of a somewhat delayed insulin initiation, especially for the upper 25% who exceeded 10.2% (88 mmol/mol) before starting on insulin. In basal insulin-initiators with complete follow-up after incident insulin exposure (*n* = 248), also with a median HbA_1c_ at baseline of 8.8% (IQR, 8.0, 10.2) (73 mmol/mol (IQR, 64, 88)), 47% and 35% reached an HbA_1c_ of <7.5% (58 mmol/mol) and <7.0% (53 mmol/mol), respectively. Patients attaining HbA_1c_<7.0% (53 mmol/mol) with insulin, as compared to non-attainers, were characterized by a slightly lower baseline HbA_1c_ (8.6% (IQR, 7.7, 9.7) (70 mmol/mol (IQR, 61, 83)) vs. 9.1% (IQR, 8.1, 10.3) (76 mmol/mol (IQR, 65, 89)) and were more likely to have ≥1 vascular complication, but had about the same age, used about the same amount of NIADs and had only a slightly longer diabetes duration (Table 2). Although a few more non-attainers vs. attainers, tried other insulin formulations, e.g. fast-acting, premixed and/or basal analogue insulins, after their first NPH exposure, still attainers vs. non-attainers displayed a larger decrease in HbA_1c_ after insulin start-up (e.g. −2.0%-point (IQR, −3.6, −1.1) [-22 mmol/mol (IQR, −40, −12)] vs. −0.9%-point (IQR, −2.1, −0.2) [-10 mmmol/mol (IQR, −23, −2)], *p < 0.01*), and consequently reached a much lower HbA_1c_ level (e.g. 6.5% (IQR, 6.0, 6.9) (48 mmol/mol (IQR, 42, 52)) vs. 7.9% (IQR,7.5, 8.6) (63 mmol/mol (IQR, 58, 70)) in non-attainers, *p < 0.01*) (Supplementary Figures 2 and 3).

**Table 2.** Descriptive analysis of the characteristics of basal insulin (NPH) initiators who did or did not attain HbA_1c_ < 7% (53 mmol/mol) (e.g. from the study sample for secondary outcome analysis).

*Study groups Key descriptive dimensions*		*Attaining* HbA_1c_<7% (*n* = 100)	*Not attaining* HbA_1c_<7% (*n* = 158)	*P*-value
***Demographics***				
Age in years (Mean ± SD)		63.8 ± 14.1	63.5 ± 13.7	0.86
Gender: Males/Females (n (%))		60 (60) / 40 (40)	88 (56) / 70 (44)	0.50
Ethnicity (n (%))	European	91 (91)	140 (89)	0.81
	African	0 (0)	3 (2)	
	Asian	8 (8)	14 (9)	
	Other or unknown	1 (1)	1 (1)	
Diabetes duration (Median, IQR)		9.5 (5.5, 14.8)	11.2 (6.5, 16.3)	0.10
***Non-insulin anti-diabetic therapy***				
NIAD usage (n (%))	Metformin	52 (52)	83 (53)	0.90
	SU	12 (12)	13 (8)	0.39
	DPP4i	14 (14)	25 (16)	0.73
	GLP-1-RA	7 (7)	12 (8)	0.86
	SGLT-2i	4 (4)	10 (6)	0.58
	TZD	0 (0)	2 (1)	0.52
***Disease control status*** (No differences in total cholesterol, HDL cholesterol, triglycerides, blood pressure, data not shown)				
HbA_1c_ at baseline (Median, IQR)	%	8.6 (7.6, 9.7)	9.1 (8.1, 10.3)	0.02
	mmol/mol	70 (60, 83)	76 (65, 89)	
BMI (Median, IQR) (kg/m^2^)		31.7 (26.8, 37.1)	31.8 (26.9, 35.1)	0.61
LDL cholesterol (Median, IQR) (mmol/l)		3.1 (2.5, 4.1)	2.9 (2.2, 3.9)	0.21
Creatinine (Median, IQR]) (μmol/l)		96.0 (76.0, 132.3)	89.0 (75.0, 128.0)	0.60
***Complications***				
*Micro-vascular diseases (n, %)*	None	72 (72)	96 (61)	0.07
	One or more	28 (28)	64 (39)	
*Macro-vascular diseases (n, %)*	None	67 (67)	95 (60)	0.26
	One or more	33 (33)	63 (40)	
***Characterization of initiated insulin therapy***				
Share with shift or add-on with non-NPH insulin prior lowest HbA_1c_ (%)		27	37	0.09
Time until change (Days)		30 (30, 173)	30 (30, 180)	0.34
Share who try fast acting insulin (%)		30	42	0.04
Share who try Pre-Mixed insulin (%)		12	15	0.55
Share who try basal insulin analogue (%)		4	3	0.50
Median number of NIADS at end of follow-up		1 (0, 1)	1 (0, 1)	0.93
Lowest attained HbA_1c_	%	6.5 [6.1, 6.9)	7.9 (7.5, 8.69	< 0.01
	mmol/mol	48 (42, 52)	63 (58, 70)	
Change in HbA_1c_	%-point	−2.0 (-3.6, −1.1)	−0.9 (-2.1, −0.2)	< 0.01
	mmol/mol	−22 (-40, −12)	−10 (-23, −2)	
Time to lowest HbA_1c_ (Weeks)		35 (22, 53)	32 (21, 52)	0.81

Abbreviations: NIAD: Noninsulin anti-diabetic drug.

Differences between categorical variables, given as percentages, were assessed by Chi-square tests if expected cell counts were > 5, if not a by Fisher’s exact. Continuous variables are given as means ± SD or medians with interquartile range (IQR). Differences in mean and medians of continuous and normally distributed variables were explored using independent samples T-test. Non-normally distributed continuous variables were assessed by Mann–Whitney U-test.

### Risk of timely versus postponed basal insulin-initiation

Diabetes nurse access, but not support staff diabetes course, related to a more than two-fold higher chance of ‘timely’ insulin-initiation both prior to and after adjustments (Table 3). Whilst the risk of timely insulin start-up increased with support staff size, this effect was only marginal (e.g. 1% increase in RRR per 1 increase in staff FTE), and further a similar numerical, but non-significant, trend was detected in the *postponed* insulin-initiation group making this latter finding difficult to interpret.

**Table 3.** The relative risk ratios (RRR) and odds ratios (ORs) for the primary and secondary outcomes are shown for each key support staff factor (exposures).

**Primary outcome analysis** Relative risk ratios (RRR) (Ref: *NIAD only treated (N = 3,781), RRR = 1)* with 95% CIs of *postponed* and *timely* basal insulin-initiation for each key exposure				
Outcomes Exposures	*Timely* basal insulin initiation (*N* = 294)		*Postponed* basal insulin-initiation (*N* = 219)	
	Crude RRR (95% CI)	Adjusted RRR (95% CI)	Crude RRR (95% CI)	Adjusted RRR (95% CI)
**Support staff diabetes competence factors**				
Clinic diabetes nurse	2.42 (1.69, 3.46)***	2.10 (1.36, 3.24)^***a^	1.06 (0.68, 1.65)	1.11 (0.65, 1.89)^a^
Support staff diabetes course (e.g. share of sup. staff with course)	1.13 (0.80, 1.59)	0.88 (0.64, 1.22)^b^	0.98 (0.67, 1.42)	0.93 (0.65, 1.32)^b^
**Support staff operational capacity factors**				
Support staff work-load (e.g. Support staff-to-GP FTE^#^ ratio)	0.96 (0.88, 1.06)	0.99 (0.91, 1.08)^c^	1.01 (0.91, 1.11)	0.97 (0.89, 1.07)^c^
Support staff size (e.g. Sum staff position sizes given in FTEs)	1.01 (1.00, 1.03)*	1.01 (1.00, 1.02)^*d^	1.01 (0.99, 1.02)	1.01 (1.00, 1.02)^d^
**Secondary outcome analysis** Odds ratios (ORs) with 95% CIs of attainment of HbA_1c_ < 7% (53 mmol/mol)				
Outcome Exposure	Do attain HbA_1c_<7% (53 mmol/mol)			
	Crude OR (95% CI)		Adjusted OR (95% CI)	
**Support staff diabetes competence factors**				
Clinic diabetes nurse	1.17 (0.62, 2.20)		0.93 (0.46, 1.89)^a^	
Support staff diabetes course (e.g. share of sup. staff with course)	1.84 (1.01, 3.36)*		1.70 (0.90, 3.22)^b^	
**Support staff operational capacity factors**				
Support staff work-load (e.g. Support staff-to-GP FTE ratio)	1.83 (0.60, 5.54)		1.41 (0.38, 5.18)^c^	
Support staff size (e.g. Sum of staff position sizes given in FTEs)	1.00 (0.99, 1.01)		1.00 (1.00, 1.01)^d^	

*Primary outcomes:* Relative risk ratios (RRR) with 95% CIs were obtained from the generalized structural equation model (GSEM) multinomial logistic regression with random effects showing key staff support factors that were associated with postponed and timely basal insulin-initiation.

*Secondary outcomes*: Table 3B. Odds ratios (ORs) and their 95% CIs were obtained from the binary logistic regression model with random effects showing factors that were associated with attainment of HbA_1c_ < 7% (53 mmol/mol).

Adjustments:.

^a^ Adjusted for: Support staff hands-on diabetes follow-up tasks (mediators), Support staff diabetes course (confounder), Support staff operational capacity factors (confounders);.

^b^ Adjusted for: Support staff hands-on diabetes follow-up tasks (mediators), Clinic diabetes nurse (confounder), Support staff operational capacity factors (confounders);.

^c^ Adjusted for: Support staff hands-on diabetes follow-up tasks (mediators), Clinic diabetes nurse (confounder), Support staff diabetes course (confounder), Total support staff FTEs (e.g. variable on support staff size) (confounder);.

^d^ Adjusted for: Support staff hands-on diabetes follow-up tasks (mediators), Clinic diabetes nurse (confounder), Support staff diabetes course (confounder), Support staff-to-GP-FTE ratio (e.g. variable on support staff work-load) (confounder).

*All adjustments were based on directed acyclic graph analysis, and no other factors were identified as potential mediators or confounders*.

*About the variables:* **Support staff-hands on diabetes follow-up tasks**: 1) staff patient support of BG measurements, a categorical variable (yes/no); 2) staff patient support of diet, a categorical variable (yes/no); 3) staff patient support of injection therapies (e.g. GLP-1-RAs and/or insulin), a categorical variable (yes/no); 4) staff usage of Noklus, a categorical variable (yes/no)***. Support staff diabetes competence factors***: 1) *Support staff diabetes course*: a continuous variable on share of staff members with a formal diabetes course within the last 3 years; 2) *Clinic diabetes nurse*: indicates presence or absence of a diabetes nurse in the GP clinic, a categorical variable (yes/no). **Support staff operational capacity factors***: 1) Support staff-to-GP FTE ratio*: a continuous variable expressing of staff work-load; 2) *Total number of support staff FTEs:* a continuous variable expressing staff size.

*#Abbreviations: FTE: abbreviation for Full-Time-Equivalent. This is defined as number of total hours worked by an individual divided by the maximum number of compensable hours in a full-time schedule as defined by law in Norway. In Norway 1 FTE, also often referred to as one 100% employment position, equals an employment of 37.5 h per week*;.

*** *p* < 0.001;.

** *p* < 0.01;.

* *p* < 0.05.

### Odds of attaining HbA_1c_ <7.0% (53 mmol/mol)

Prior to adjustments, share of support staff with diabetes course related a higher risk of attaining HbA_1c_ <7.0% (53 mmol/mol) after basal insulin-initiation (Table 3) (OR = 1.84, [95%CI, 1.10, 3.36]), but after adjustment, this was no longer statistically significant. Diabetes nurse and factors indicatory of support staff operational capacity were found unrelated to the risk of attaining glycemic targets.

## Discussion

### Statement of principal findings

In the present study, we assessed the quality of basal insulin initiation in individuals with T2D cared for in Norwegian general practice. Insulin therapy was instituted at a median HbA_1c_ of 8.8% (IQR, 8.0, 10.2) [73 mmol/mol (IQR, 64, 88)] which after 34 weeks declined by a median 1.4%-point (IQR, −2.7, −0.6) [-15 mmol/mol (IQR, −30, −7)] whereby 35% attained an HbA_1c_ <7% (53 mmol/mol). The rather high HbA_1c_ at baseline, where 25% of all patients exceeded a value of 10.2% (88 mmol/mol) before starting on insulin, further indicates that insulin initiation was very susceptible to therapeutic inertia. Moreover, the likelihood of timely basal insulin initiation (primary outcome), but not attainment of HbA_1c_<7% (53 mmol/mol) with insulin (secondary outcome), increased more than twofold if access to a diabetes nurse. In contrast, no other staff factors with relevance for diabetes follow-up related significantly to neither the primary nor the secondary outcome.

### Strengths and weaknesses of the study

While the sample sizes for the primary outcome were considered sufficient, however, the sample sizes for the secondary outcome analyses, partly due to incomplete follow-up on 49 out of 307 basal insulin initiators, may have been too small for us to be able to draw firm conclusions.

Another potential limitation to the current study is that it builds on data from the years 2012-14. But although clinical practice in some aspects has changed dramatically since then, especially due to the increased usage of glucagon-like-polypeptide 1(GLP-1)-receptor agonists and sodium-glucose cotransporter-2 (SGLT2)-inhibitors which may postpone the need for insulin, still the organization of the Norwegian general practice has not changed much since then, the national guidelines still communicate the same overall glycemic target, and NPH insulin is still the mandatory first choice when initiating basal insulin in Norway. Further, a key strength of the present study is that the ROSA 4 dataset is characterized by very good representativeness for Norwegian general practice [18]. As a consequence, we also think the present ROSA 4-samples, identified and analyzed to study how support staff factors may affect basal insulin initiation in Norwegian general practice, are valid and still relevant for the current clinical practice in Norway.

Yet another limitation is that the ROSA4 dataset does not include data on potentially very important GP dimensions such as the overall patient list size, the GPs personal attitudes and beliefs in relation to insulin initiation [10], GP attendance to relevant postgraduate courses or training in insulin initiation and treatment [21], and the overall GP continuity of care [22]. Thus, although we found no significant relation between GP factors and the risk of *timely* or *postponed* basal insulin-initiation in our pilot analyses (Supplementary Table 1), this does not rule out that the GP also has an important part to play. Hence, future studies on more comprehensive datasets, capable of characterizing some of the above-mentioned key dimensions of the GP, are needed to be able to fully understand the potential role of intrinsic GP factors in relation to the quality of insulin initiation in Norwegian general practice.

In our directed acyclic graphs (data not shown) carried out prior to analyses based on the defined exposures and outcomes, all patient factors were identified as only ‘nonconfounders for the outcomes’, and hence were not adjusted for. However, the current data set did not include socio-economic factors. Especially self-injection, diet counseling and blood glucose self-measurements are measures that are likely to be more challenging to carry out in patients with low socioeconomic status. Thus, it still remains to be solved whether patient socio-economic status may potentially modulate the effect of a diabetes nurse on the chance of timely insulin initiation.

### Comparisons to other studies

A Danish real-life study on basal insulin-initiation in individuals with T2D reported a median HbA_1c_ of 9.2% at baseline, and median decline of 1.6% with basal insulin by which 29% attained HbA_1c_ <7% [23]. Hence, they reported a little higher HbA_1c_ at baseline, and despite of a larger decline in HbA_1c_, fewer subjects reached HbA_1c_<7% *(53 mmol/mol)* as compared to our study. However, their follow-up period after insulin initiation was only ∼26 weeks, thus substantially shorter than ours. In addition, some of the subjects included in the Danish study may have been initiated at a specialist outpatient clinic, possibly explaining the higher HbA_1c_ at baseline but also the shorter treatment time needed to attain target. For further comparison, a UK retrospective database study reported only 17% reaching HbA_1c_<7% after starting insulin, but those subjects also had higher HbA_1c_ at baseline and were given only ∼17 weeks of follow-up, e.g. only about half of ours. Thus, we think our sample of basal insulin-initiators seems both representative and valid in relation to previous observations.

Access to a diabetes nurse, but not support staff with diabetes course, increased the likelihood of *timely* basal insulin-initiation. This is a new finding and persisted after adjustments for confounders. This suggests that the diabetes nurse seems to offer unique clinical benefits to the care which cannot be fully replicated by other type of clinic support staff, despite relevant hands-on involvement or training. Such notion is further supported by another general practice study, reporting higher insulin initiation-rates and better glycemic control with an intervention with a practice nurse who, assisted by a nurse with formal diabetes competences, led all insulin initiations [24].

### Meaning of the study

A previous study reported that GPs generally tend to accept higher HbA_1c_ levels than endocrinologists before starting insulin treatment [10]. In support of that notion, despite Norwegian guidelines clearly recommend an HbA_1c_ <7.0% (53 mmol/mol) for most patients, HbA_1c_ was allowed to reach a median of 8.8% (IQR, 8.0, 10.2) (73 mmol/mol (IQR, 64, 88)) before basal insulin-treatment was initiated. Moreover, only 47% and 35% of insulin initiators with complete follow-up reached an HbA_1c_ of <7.5% (58 mmol/mol) and <7.0% (53 mmol/mol), respectively. Thus, our Norwegian general practice data suggest both basal insulin initiation and titration may be affected by therapeutic inertia. In further line with these considerations, some may argue that the chosen HbA_1c_- cut-off level used by our algorithm (STEP5A and 5B, Figure 1) to identify patients with *postponed* basal insulin-initiation should have been much lower. Although we fully acknowledge these considerations, still we think the employed inclusion algorithm is in good agreement with the prevailing modus operandi in Norwegian general practice. Further, we believe the current group identified with *postponed* basal insulin-initiation, despite the potential exclusion of subjects referred to an outpatient diabetes clinic, still represents a clinically relevant sample of patients highly affected by therapeutic inertia. This group, apart from being more hyperglycemic and on more non-insulin anti-diabetic drugs (NIADs), were younger, more likely to be males and with Asian ethnic background, and less likely to have vascular complications as compared to those with *timely* insulin-initiation (Table 1). Although our findings seem to indicate that male gender, Asian ethnic minority background and/or young age may increase the risk of therapeutic inertia and delayed insulin-initiation in Norwegian general practice, further studies are needed to confirm this notion.

Neither access to a diabetes nurse nor other support staff factors with relevance for diabetes follow-up related to the chance of reaching HbA_1c_ <7.0% (53 mmol/mol) with insulin. Whilst support staff diabetes course was significantly related to HbA_1c_<7.0% *(53 mmol/mol)* in the unadjusted analysis, this relation did not persist after adjustments. Although this implies that the diabetes nurse may be less involved in insulin titration than in insulin initiation, we must keep in mind that the power may be too small for the secondary outcome analyses to draw firm conclusions. Additional studies are therefore needed to disclose how a diabetes nurse may affect the quality of insulin titration and intensification within a Norwegian general practice setting.

While Slåtsve et al. [25] also found a positive impact of having a diabetes nurse on the quality of glycemic control in Norwegian primary care patients with T2D, in addition, they also demonstrated an independent positive effect of using the NOKLUS diabetes follow-up form. Correspondingly, although our data seem to suggest that training and involvement of an ordinary support staff has little effect on the quality of insulin initiation, still our findings do not rule out that such measures, including involving support staff in NOKLUS usage, may have a positive effect on other quality measures of diabetes treatment. More studies are however needed to determine how training and involvement of an ordinary practice support staff care should look like to be of potential value and relevance for the current diabetes care.

Although the diabetes nurse was found to have a positive effect on *timely* basal insulin-initiation, this was not the case for the risk of *postponed* basal insulin-initiation. This discrepancy, however, may at least partly be ascribed to the design of our study where some subjects, in spite of being detected with incident NPH insulin-exposure, were excluded from the sample because the GP medical files lacked information on the preceding HbA_1c_-levels (Figure 1, STEP 3 A). It is likely that some of these excluded individuals instead started insulin treatment at a specialist outpatient clinic, a measure that would require a GP referral but also postpone the start-up. Individuals with *postponed* as compared to *timely* insulin start-up, per definition, due to the HbA_1c_- and treatment-based selection algorithm, were more hyperglycemic and were found with HbA_1c-_levels still rising until the very end of the follow-up despite much higher usage of NIADs. For such people, according to our data, a practice diabetes nurse could have been highly beneficial by facilitating an earlier initiation of insulin treatment and thus attainment of better glycemic control.

The majority of GP clinics with access to a diabetes nurse were located in the Nordland county (Supplementary Table 2). This marked regional difference was however much expected as Nordland since the beginning of last decade have invested vastly in primary care diabetes nurses assisting local general practices. In line with our findings, Nordland county also had 43.1% more patients than expected (*p* < 0.01 at Chi-square tests (full analysis not shown)) with “timely insulin initiation”.

The current finding that diabetes nurse access increases likelihood of timely insulin initiation in general practice may be mediated by multiple factors that we were not able to account for in the present study, such as the frequency and duration of 1-on-1 diabetes consultations, and the content and extent of the provided patient counselling and training. Thus, future studies are needed to determine what the underlying possible key mediating factors may be.

In conclusion, in Norwegian general practice patients with T2D, both basal insulin-initiation and –titration may be affected by therapeutic inertia. However, access to a practice diabetes nurse increased the chances of *timely* basal insulin-initiation. This effect could not be replicated by an ordinary type of support staff regardless of total support staff size or support staff size relative to total number of GPs, and regardless of whether the support staff had been provided with a diabetes course and/or were hands-on involved in diabetes follow-up. Our data provides additional support the notion that a diabetes nurse vs. an ordinary clinic support staff may provide unique benefits to diabetes care in Norwegian general practice.

## Supplementary Material

Supplemental Material

Supplemental Material

## Notes

1 NOKLUS: “The NOKLUS diabetes form” which was launched in 2008, and after that time was gradually implemented in most general practice electronic patient record systems in Norway. The form collects national data on the quality of diabetes treatment in Norway, it is well suited for carrying out an annual diabetes control in a structured manner, it has been shown to associate to better quality of care and it’s usage is strongly recommended by Norwegian health authorities. NOKLUS includes the following variables: diabetes type and year of diagnosis, HbA1c, blood pressure, LDL cholesterol, possible history and status of retinopathy and nephropathy, possible history of CHD, stroke, limb amputations and foot ulcers, monofilament tests, foot pulse status, smoking status and history, hypoglycemia frequency, body weight, height, Body Mass Index (BMI), driver’s license status, frequency of retinopathy screening, and current anti-diabetic, anti-hyperlipidemic and anti-hypertensive medications (https://www.noklus.no/norsk-diabetesregister-for-voksne/variabeloversikt/).

2 FTE: abbreviation for Full-Time-Equivalent. FTE is computed as number of total hours worked by an individual employee divided by the maximum number of compensable hours in a full-time schedule as defined by law in Norway. In Norway 1 FTE, also often referred to as one 100% employment position, equals an employment of 37.5 hours per week.
