# Supplementary material for: Insulin initiation in patients with type 2 diabetes is often delayed, but access to a diabetes nurse may help—insights from Norwegian general practice
Source: Scand J Prim Health Care. 2024 Feb 7;42(1):132–43. doi: 10.1080/02813432.2023.2296118 (PMC10851798; doi:10.1080/02813432.2023.2296118)
Supplement: Supplemental Material [file IPRI_A_2296118_SM7052.docx]

Supplementary figure 1.


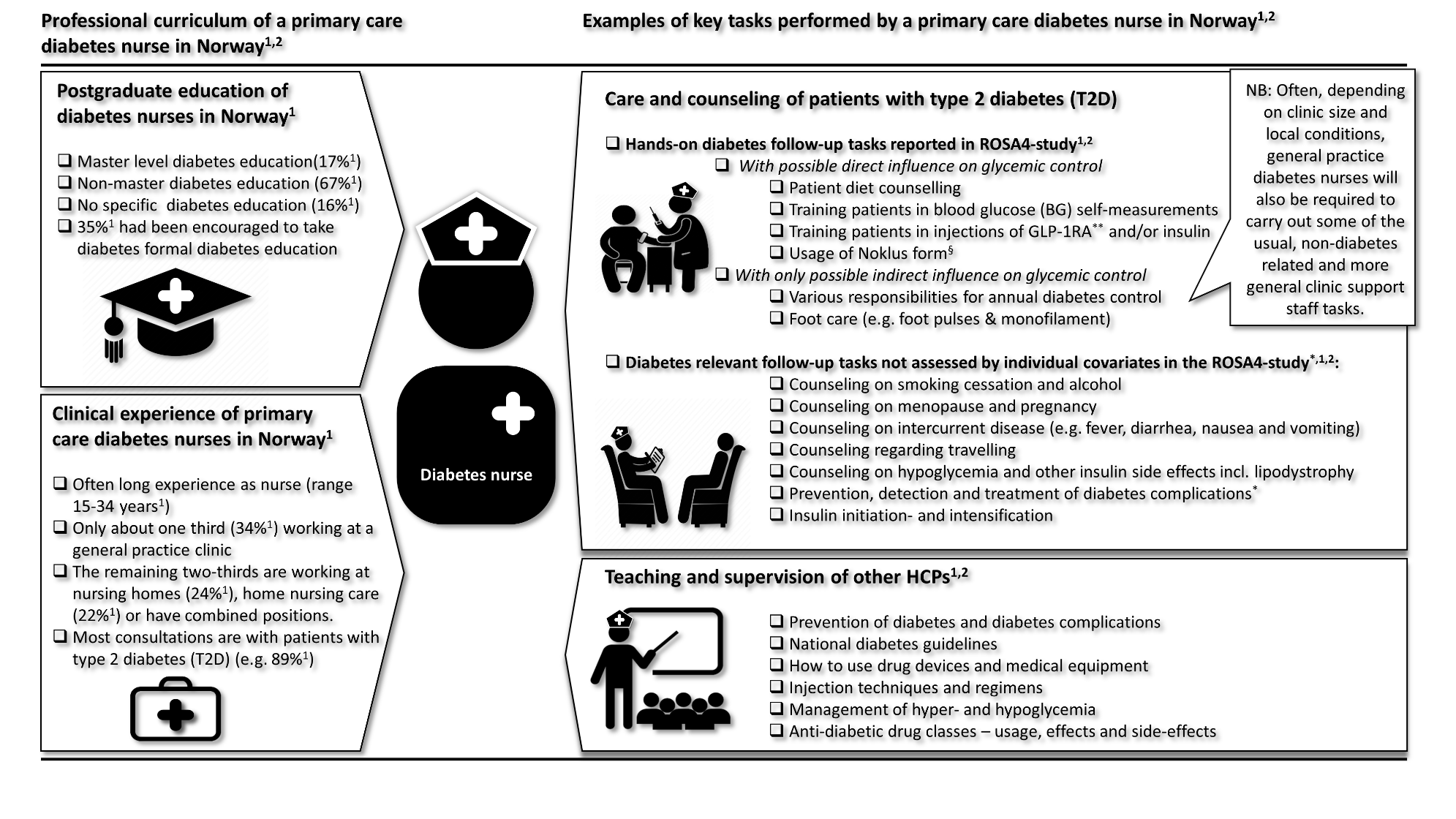
Overview of the usual professional curriculum as well as the most important key deliverables of a primary care diabetes nurse in Norway^1,2^

^1^Kollveit BC H et al. HVL-rapport nr. 18 2021, Høgskulen på Vestlandet (HVL), 2021 (Title: Diabetessykepleiers ansvars- og funksjonsområder i helsetjenesten); ^2^Authors own clinical experience and insights from discussions with colleagues working within Norwegian primary care and general practice; ^*^Not included as covariates in the ROSA4 study sample; ^**^GLP-1-RA: Glucagon-Like-Peptide 1 Receptor Agonist; ^§^The Noklus diabetes form is an electronic tool that interacts with the record system. The form is well suited when carrying out an annual diabetes control, and it’s usage is strongly recommended by Norwegian health authorities. HCP: Health care personnel

Supplementary figure 2.


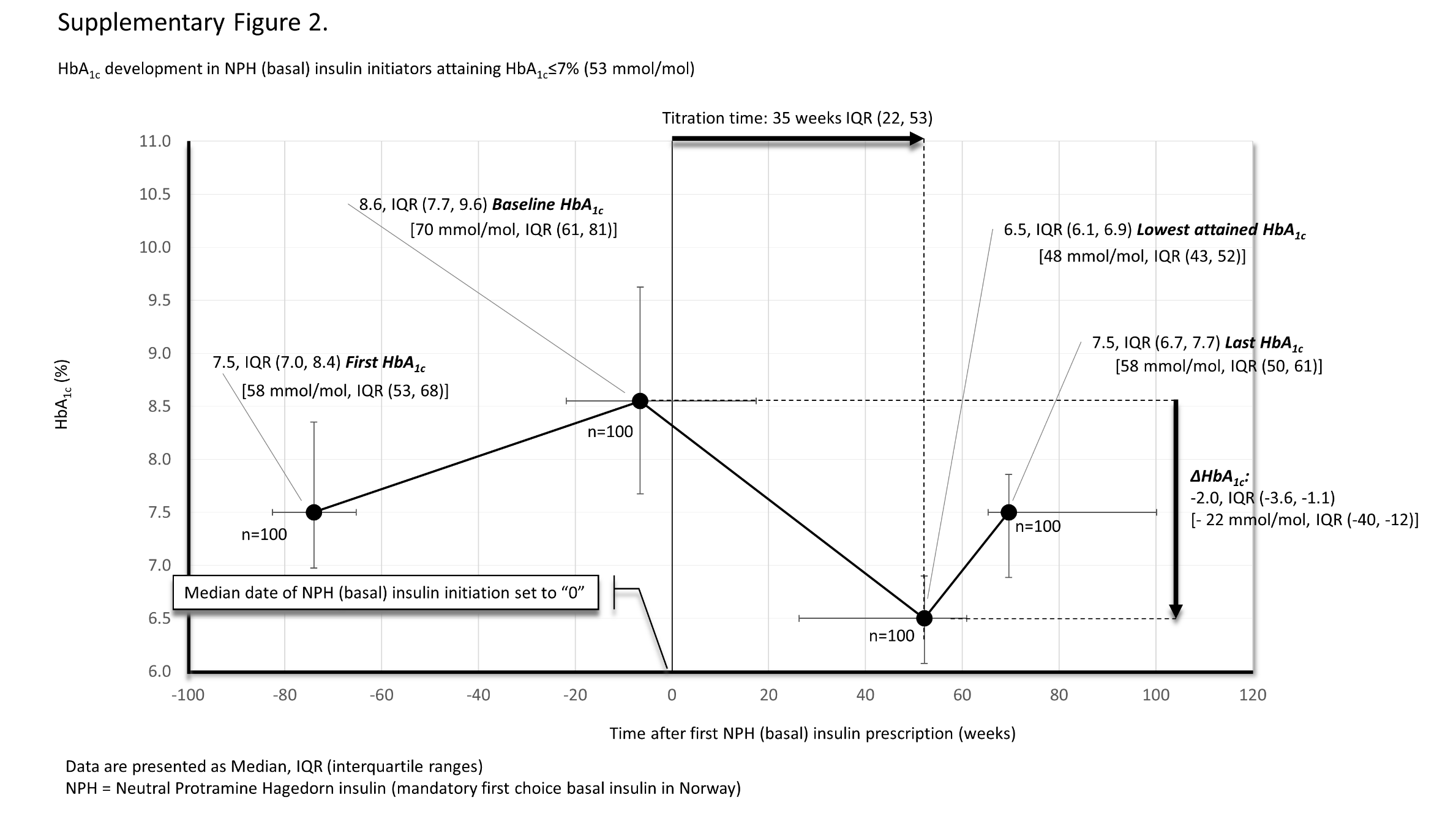
HbA_1c_ development in basal insulin (NPH)-initiators attaining HbA_1c_<7% (53 mmol/mol).

Supplementary figure 3.


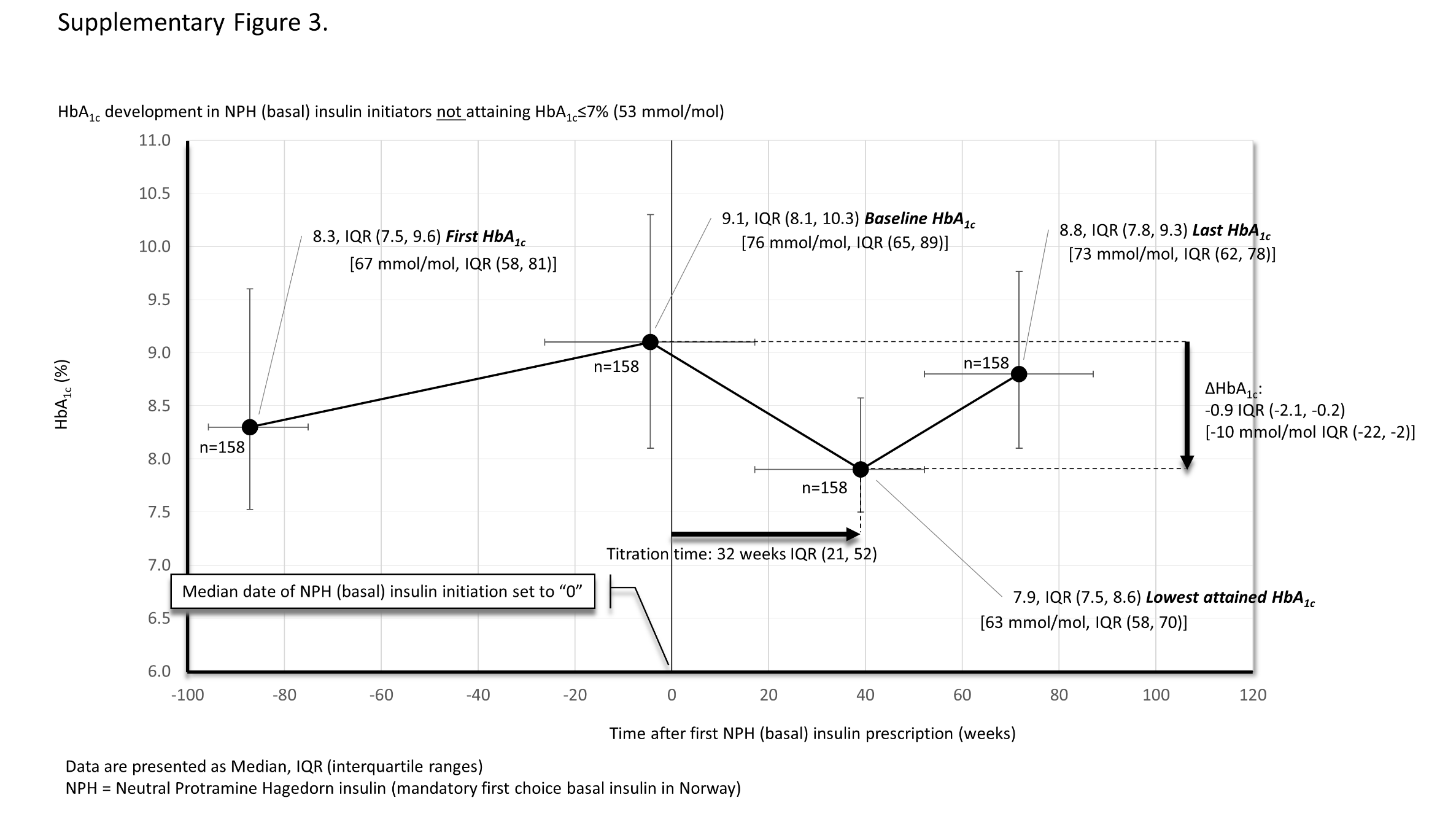
HbA_1c_ development in basal insulin (NPH)-initiators not attaining HbA_1c_<7% (53 mmol/mol).
