# Supplementary material for: Insulin initiation in patients with type 2 diabetes is often delayed, but access to a diabetes nurse may help—insights from Norwegian general practice
Source: Scand J Prim Health Care. 2024 Feb 7;42(1):132–43. doi: 10.1080/02813432.2023.2296118 (PMC10851798; doi:10.1080/02813432.2023.2296118)
Supplement: Supplemental Material [file IPRI_A_2296118_SM7051.docx]

**Supplementary Table 1.** Pilot analysis of risk of *postponed* vs. *timely* basal (NPH) insulin-initiation by GP factors (NB: *NIAD^*^ only-treatment* is the reference group (e.g. with RRR = 1) (data not shown))

| Outcome  Covariates | **Timely basal insulin-initiation** | | **Postponed basal insulin-initiation** | |
| --- | --- | --- | --- | --- |
|  | RRR (95% CI) | *P*-value | RRR (95% CI) | *P*-value |
| **GP level factors** | | | | |
| Age in years | 1.00 (0.99, 1.01) | 0.73 | 1.01 (0.99,1.02) | 0.26 |
| GP gender (ref: Male) |  |  |  |  |
| Female | 0.96 (0.74, 1.24) | 0.77 | 0.90 (0.66, 1.22) | 0.51 |
| GP specialist (yes/no) | 0.92 (0.69, 1.23) | 0.57 | 1.06 (0.76, 1.48) | 0.71 |
| Years in primary care | 1.00 (0.99, 1.01) | 0.56 | 1.00 (0.99, 1.02) | 0.66 |
| Time since authorization | 1.00 (0.99, 1.01) | 0.53 | 1.01 (0.99, 1.02) | 0.39 |
| Experience in Norway (years) | 1.01 (1.00, 1.01) | 0.19 | 1.00 (0.99, 1.01) | 0.81 |

^*^NIAD: Non-insulin anti-diabetic drug.

Unadjusted relative risk ratios (RRR) with 95% Cis were obtained from logistic regression. Relative risk ratios for the third nominal outcome, NIAD only, are not shown.

**Supplementary Table 2.** Descriptive overview of the characteristics of the GP and practice clinic processes and routines.

| **GP characteristics (n =275)** | | |
| --- | --- | --- |
| *Key GP descriptive parameter* | | |
| Age in years (Median, IQR) | | 51.0 (40.0, 60.0) |
| Number of years as a GP in primary care (Median, IQR) | | 18.0 (7.0, 29.5) |
| Number of years in Norway (Median, IQR) | | 45.0 (34.0, 58.0) |
| Number of DM patients per GP (Median, IQR) | | 37.6 (30.2, 43.5) |
| Number of HbA_1c_ measurements per year (proxy for number of diabetes consultations) (Median, IQR) | | 40.0 (21.3, 62) |
| ^1^Number of GPs by gender [n (%)] | | |
| Males | | 147 (53.5) |
| Females | | 123 (44.7) |
| Number of GPs by county [n (%)] | |  |
| Oslo | | 51 (18.5) |
| Akershus | | 48 (17.5) |
| Hordaland | | 41 (14.9) |
| Rogaland | | 65 (23.6) |
| Nordland | | 70 (25.5) |
| Specialist in general practice (Yes) [n (%)] | | 186 (67.6) |
| GPs sharing patient list with other doctor (Yes) [n (%)] | | 21 (7.6) |
| GPs who use NOKLUS (incl. where usage is delegated to support staff/diabetes nurse) (Yes) [n (%)] | | 150 (54.5) |
| **Practice clinic organization (n = 74)** | | |
| *Key clinic parameter* | |  |
| Number of GPs per clinic (FTEs) (Median, IQR) | | 3.98 (2.61, 5.01) |
| Support staff size (FTEs) (Median, IQR) | | 3.00 (2.00, 4.00) |
| Number of DM patients/ practice (clinic) (Median, IQ) | | 136.5 (84.5, 208.5) |
| GP age (years) (Median, IQR) | | 50.0 (43.2, 57.6) |
| GP clinical experience (years) (e.g. total time working as a doctor) (Median, IQR) | | 19.3 (11.7, 27.3) |
| GP clinical experience as GP (years) (e.g. time spent working as GP ) (Median, IQR) | | 19.0 (10.1, 27.0) |
| Clinic DM patient/ GP FTE ratio (e.g. number of diabetes patients per GP) (Median, IQR) | | 36.1 (29.0, 43.2) |
| Number of HbA_1c_ measurements per year (proxy for number of diabetes consultations) (Median, IQR) | | 130 (78, 251) |
| DM patients to support staff-FTE ratio (e.g. number diabetes patients per members of support staff) | | 47.6 (36.9, 59.2) |
| Support staff to GP FTE ratio (e.g. staff-to-GP coverage, a proxy for staff-work-load) (Median, IQR) | | 0.8 (0.6, 0.9) |
| Support staff (regardless of individual position size) with a diabetes course within last 3 years [n (%)] | | 20.8 (34.8) |
| Clinic diabetes nurse (yes) [n (%)] | | 16 (21.6) |
| - By participating county | |  |
| - Akershus (in total 10 participating clinics) | | 1 (10.0) |
| - Hordaland clinics (in total 10 participating clinics) | | 1 (10.0) |
| - Nordland (in total 23 participating clinics) | | 13 (56.5) |
| - Oslo (in total 11 participating clinics) | | 0 (0.0) |
| - Rogaland (in total 20 participating clinics) | | 1 (5.0) |
| Support staff involvement in ROSA4 hands-on diabetes follow-up tasks (yes) [n (%)] | | |
| - Hands-on tasks with possible direct influence on glycemic control and study outcomes | Patient blood glucose self-measurement training | 28 (37.8) |
|  | Patient training in self-injection (GLP-1 RA/ insulin) | 17 (23.0) |
|  | Patient diet counselling | 32 (43.2) |
|  | Using NOKLUS form at diabetes follow-ups | 23 (31.1) |
| - Hands-on tasks without immediate or direct influence on glycemic control and study outcomes | Patient foot care | 17 (23.0) |
|  | Other undefined tasks in relation to annual diabetes control | 7 (9.5) |

Statistics given are either n (%) or median (Q1, Q3). ^1^Five GPs did not reveal their gender

Supplementary Table 3A.


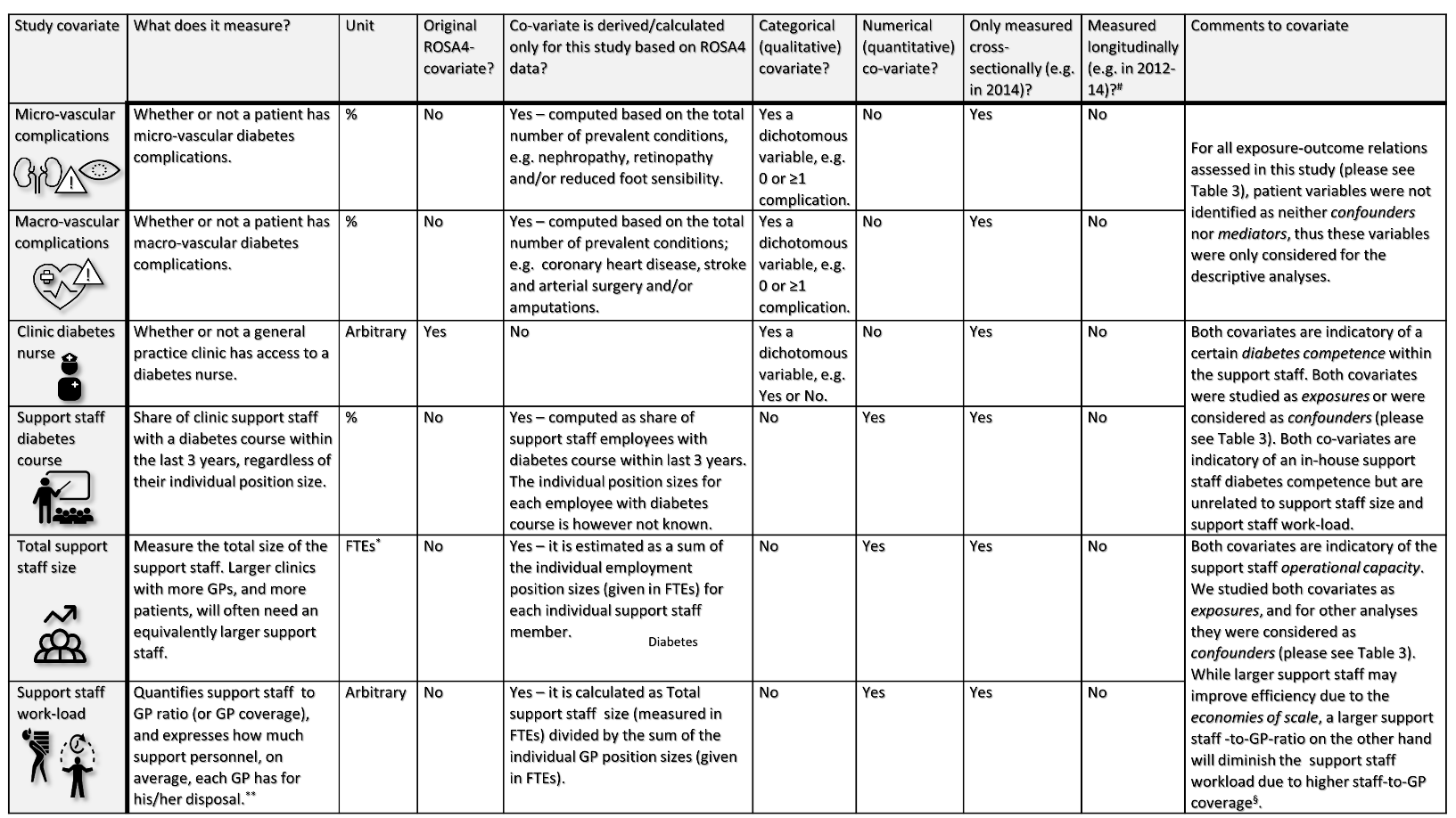
 Additional details for a selected study co-variates which may require further clarification.

^*^FTE: abbreviation for Full-Time-Equivalent. It is here defined as number of total hours worked by an individual employee divided by the maximum number of compensable hours in a full-time schedule as defined by law in Norway. In Norway 1 FTE, also often referred to as one 100% employment position, equals an employment of 37.5 hours per week.

^**^In Norway, the average SS- to-GP ratio (or support staff GP coverage) is 0.8^*^according to data from 2017, from the Norwegian Medical Association (NMA) (<https://www.legeforeningen.no/foreningsledd/lokal/troms/skalpellen/2017/losning-doble-per-capita-opp-til-1000-pasienter/>). Thus, the present sample is quite comparable to this finding.

^#^Longitudinal data: Longitudinal data: Only HbA_1c_ and drug prescriptions were recorded longitudinally, and primarily for year 2012-14 although in some cases also for year 2015 and 2016. All other study variables were cross-sectional from year 2014.

^§^When we use the support staff-to-GP ratio as a proxy for support staff work-load we at the same time assume each GP full time position will generate the same work-load for the clinic support staff, although this may not be entirely true as this may depend on patient list demography, patient list size and usage of laboratory services etc.

Supplementary Table 3B.


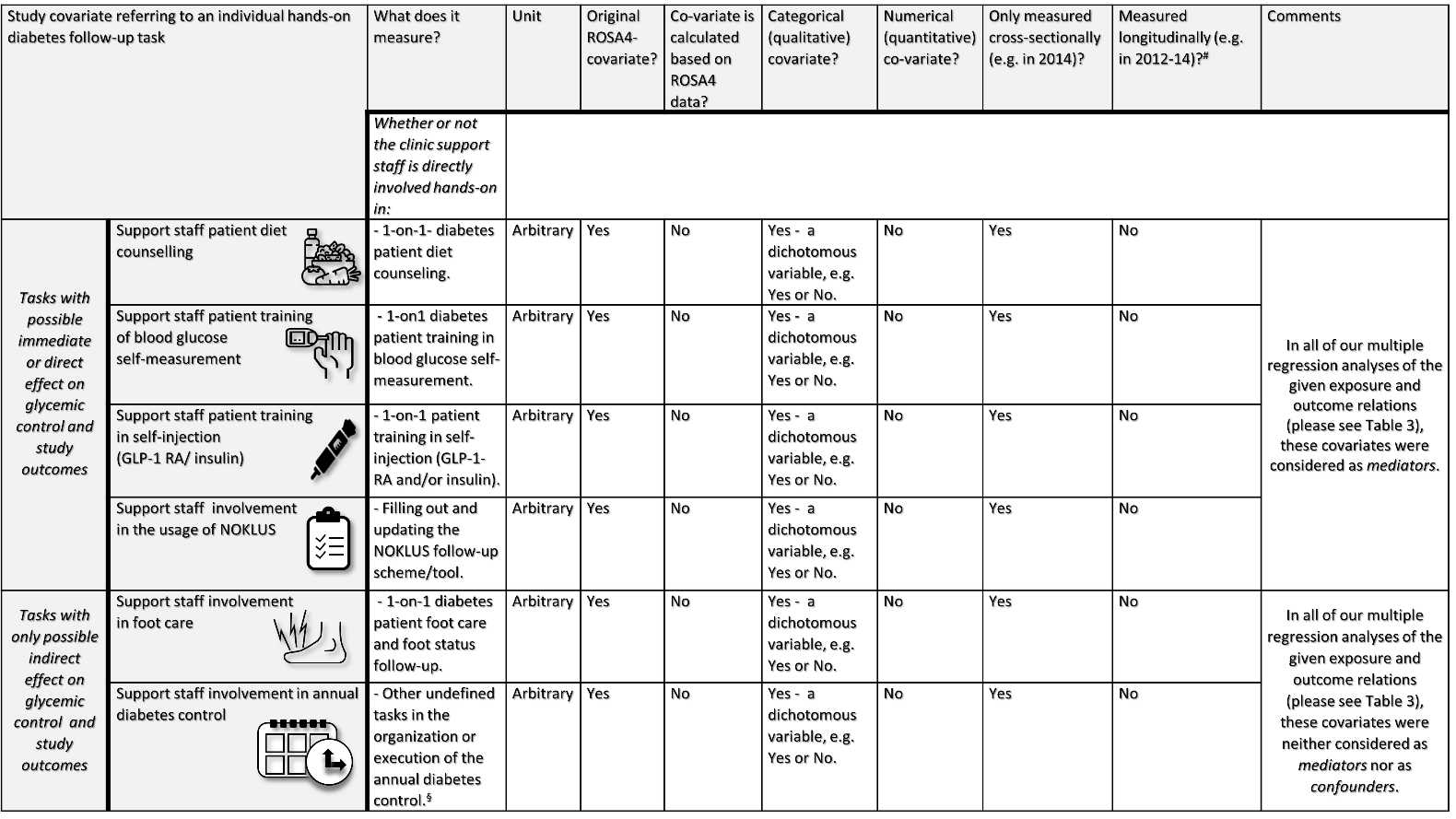
Additional details on the ROSA4-hands-on diabetes follow-up tasks, co-variates which may require further clarification.

Abbreviation: GLP-1-RA: Glucagon-like-peptide 1 receptor agonist.

^*^FTE: abbreviation for Full-Time-Equivalent. It is here defined as number of total hours worked by an individual divided by the maximum number of compensable hours in a full-time schedule as defined by law in Norway. In Norway 1 FTE, also often referred to as one 100% employment position, equals an employment of 37.5 hours per week.

^**^In Norway, the avg. support staff to GP ratio (or support staff GP coverage) is 0.8^*^according to data from 2017, from the Norwegian Medical Association (NMA) (<https://www.legeforeningen.no/foreningsledd/lokal/troms/skalpellen/2017/losning-doble-per-capita-opp-til-1000-pasienter/>)

^#^Longitudinal data: Longitudinal data: Longitudinal data: Only HbA_1c_ and drug prescriptions were recorded longitudinally, and primarily for year 2012-14 although in some cases also for year 2015 and 2016. All other study variables were cross-sectional from year 2014.

^§^Although indicatory of other ways in which SS may support the annual diabetes control, the exact nature of such «additional» support is unfortunately unclear, it was not related to the assessed study outcomes in the pilot study (data not shown) and hence this covariate was not considered as mediator (or confounder) for the exposure and outcome relations analyzed and displayed in Table 4.

**Supplementary information regarding study group inclusion algorithm (as shown in Figure 1):**

**STEP 4A (please see Figure1) – Basal (NPH) insulin-initiators**

*Key inclusion criteria:* Zero insulin prescription for ≥360 days prior to first NPH exposure, with ≥1 insulin re-prescription per 360 days of follow-up after insulin start-up (e.g. to exclude patients trying but not staying on insulin), and with a baseline HbA_1c_ of ≥7.0% (53 mmol/mol) within 0-90 days prior to incident NPH insulin exposure. The latter to avoid inclusion of patients already started on insulin by other parties (e.g. specialist) and patients initiated with insulin for other reasons than failure of glycaemic control while on a Non-Insulin Anti-Diabetic (NIAD) drug regimen (e.g. patients with transient hyperglycaemia due to intercurrent disease or patients starting with basal insulin due intolerance to one or more NIAD drugs). We found 307 patients with incident basal (NPH) insulin therapy, of which 248 had complete follow-up HbA_1c_ measurements after insulin start-up.

**STEP 5A (please see Figure 1) – Basal (NPH) insulin-initiators with too late insulin start-up**

*Key criteria:* For ≥180 days prior to the date of baseline HbA_1c_, recorded with HbA_1c_ levels of ≥8 % (64 mmol/mol) or ≥9 % (75 mmol/mol) while on stable treatment with ≥3 or ≥2 different NIADs, respectively, with stable treatment defined as ≥1 NIAD prescription per 360 days recorded from 180 to 720 days (e.g. a period of 540 days) prior to baseline HbA_1c_. Only 13 patients (4.2%) fulfilled this criteria, the remaining individuals with incident insulin therapy (n=294) constituted the study group with *“timely basal insulin initiation”*.

**STEP 5B (please see Figure 1) – individuals on NIAD only where basal insulin-initiation is long overdue:**

*Key criteria:* For ≥180 days prior to last measured HbA_1c_ in 2014, found with HbA_1c_ levels above ≥8 % (64 mmol/mol) or ≥9 % (75 mmol/mol) while being on stable treatment with ≥3 (n=87) or ≥2 (n=119) different NIADs, respectively, with stable treatment defined as ≥1 NIAD prescription per 360 days recorded from 180 to 720 days (e.g. a period of 540 days) prior to last HbA_1c_-measurement in 2014. 4.9% of all NIAD exposed individuals met those requirements (n=206) and were pooled with the 13 basal insulin-initiators identified with too late insulin start-up to constitute the second study-group entitled: *“Postponed basal insulin-initiation”* (n=219). The remaining *NIAD only treated* subjects (n=3,781) formed the third nominal study group entitled: “*NIAD only treated”*. Patients not eligible for insulin initiation, e.g. patients previously exposed to insulin for other reasons (n=1,647) or on diet only (n=4,232), were excluded from this study.
